# Assessment of Selected Immune Parameters in Patients Undergoing Cardiac Surgery with the Use of Cardiopulmonary Bypass: Aspects of Age and Sex—A Pilot Study

**DOI:** 10.3390/biomedicines11041224

**Published:** 2023-04-20

**Authors:** Piotr Sindera, Ewa Kucewicz-Czech, Grażyna Wilczek

**Affiliations:** 1 Department of Molecular Biology and Genetics, Faculty of Medicine, Academy of Silesia, 40-555 Katowice, Poland; 2 Department of Cardiac Anesthesia and Intensive Care, Medical University of Silesia, 40-752 Katowice, Poland; 3 Institute of Biology, Biotechnology and Environmental Protection, University of Silesia, 40-007 Katowice, Poland

**Keywords:** cardiopulmonary bypass, infection, IL-6, immunoglobulin, aging

## Abstract

The present study aimed to assess the changes in the immunological parameters of patients undergoing cardiac surgery with cardiopulmonary bypass (CPB). The serum or plasma samples of patients were assessed to determine the concentrations of IL-6, one of the major proinflammatory cytokines (seven females and six males), and selected classes of immunoglobulins (six females and seven males). The samples for ELISA (enzyme-linked immunosorbent assay) were collected from patients before the use of CPB, at 60 min of the use of CPB, and at 24 h after the surgery. After 24 h of the surgery, IL-6, IgM, and IgG concentrations were higher in the serum of female patients than in the serum of male patients. However, compared to female patients, male patients showed a significant increase in IgG3 concentration after 24 h of the surgery. Regardless of age, the levels of the analyzed classes of immunoglobulins were similar in all patients. Additionally, in both age groups, a significant increase in the serum IL-6 concentration was observed after the first postoperative day, and this increase was more pronounced in patients diagnosed to have postoperative infections. The serum IL-6 concentration can serve as a potential marker of pathogenic infections in patients undergoing cardiac surgery with CPB and is thus useful for the early diagnosis of postoperative infections.

## 1. Introduction

Cardiopulmonary bypass (CPB) is a technique widely used in cardiac surgery to enable a temporary cardiac arrest. It supports or takes over the functions of the heart and lung during cardiac surgery [1,2]. The use of CPB is considered advantageous in cardiac operations; however, it can cause several nonphysiological responses. Although the mechanisms that contribute to a reduction in the number of immune cells after the use of CPB remain unclear, blood cell dysfunction related to CPB can be caused by various factors, such as physical damage of morphotic elements by roller pumps used for extracorporeal perfusion, blood dilution, and the flow of the body fluids in intravascular and extravascular spaces [3,4]. Interestingly, several authors have demonstrated that immune response abnormalities observed in patients due to CPB were associated with the degradation of complement system elements and an increased concentration of proinflammatory cytokines [5,6,7,8,9]. Furthermore, Scott et al. reported that the damage of morphotic elements resulting from the use of CPB perfusion devices led to a need for blood transfusion during operations [2].

Exposure of a patient’s blood to nonphysiological CPB elements may induce a generalized inflammatory reaction called Systemic Inflammatory Response Syndrome (SIRS) [10]. SIRS is a complex condition that involves humoral and cellular immune responses, including the activation of a complement cascade and a coagulation system as well as endothelial cells, leukocytes, and platelets. The use of CPB may promote the release of pro- and anti-inflammatory mediators into the blood and, more importantly, disturb their balance [11,12,13]. The increased release of cytokines may affect the functions of the respiratory system and the kidneys, or lead to myocardial damage or neurological disorders [14,15].

IL-6 is a cytokine responsible for systemic inflammation that accompanies cardiac surgeries, especially those performed using the CPB technique [16]. It is also involved in directing immune responses toward humoral reactions [17]. IL-6 production is usually increased between the third and fourth hour of a surgery [18,19,20]. A putative correlation exists between the increase in the level of cytokines in peripheral blood during the use of CPB and the subsequent organ dysfunctions. IL-6 primarily acts on B lymphocytes by stimulating their terminal differentiation into plasma cells that in turn produce immunoglobulins. Moreover, IL-6 stimulates T lymphocytes to produce IL-2 as well as synthesize its receptor and, together with IL-1, activates T cells that recognize antigens. IL-6 may also regulate the activation of resting T cells by monocytes. During SIRS, the concentration of IL-6 in serum may increase up to 100-fold; hence, this cytokine is considered an early, sensitive but nonspecific marker of bacterial infections and SIRS [21].

Immunoglobulins produced by plasma cells selectively interact with antigens, which results in the opsonization of pathogens, the activation of a complement system, antibody-dependent cytotoxicity reactions, bacteriostatic effects, and the neutralization of toxins and viruses. The use of the CPB technique has been shown to cause fluctuations in the serum levels of antibodies such as IgG1, IgG2, IgG3, IgG4, and IgA, both during operations and in the first few days of the postoperative period [22].

Immune response changes and confrontation with bacterial pathogens often delay and complicate the recovery of aged patients [23,24]. At present, there are no comparative data available referring to the level of multiple immunoglobulins before and after the use of CPB, especially in terms of the age and gender of patients. Therefore, this study aimed to compare the changes in selected immunological parameters in patients undergoing cardiac surgery with the use of CPB depending on sex and age. The kinetics of the selected immunological parameters were determined during and immediately after the surgery, with particular attention paid to elderly patients (i.e., those over the age of 70 years). In addition, the concentrations of IL-6 and various immunoglobulins (IgG, IgM, IgD, IgE, IgA, and IgG3) were determined in the serum of patients diagnosed with postoperative infections and compared to those of patients who had no signs of infection.

## 2. Materials and Methods

### 2.1. Legal Aspects

All analyses were conducted in accordance with the Polish and European regulations and approved by a local ethics committee of Medical University of Silesia (KNW/0022/KB1/164/11, approval date: 11 October 2011). Patients gave their informed consent for participation in the research study. Strictly defined inclusion criteria were applied to obtain a homogenous group of patients.

### 2.2. Inclusion and Exclusion Criteria

All included patients were subjected to a planned cardiac surgery with the use of CPB, such as replacement/angioplasty of heart valves or coronary artery bypass. Additional selection criteria applied were ejection fraction >40% and EuroSCORE ≤ 12. Exclusion criteria included infection, chronic renal failure, anemia, peptic ulcer disease, atherosclerosis of carotid arteries, chronic obstructive pulmonary disease, glycated hemoglobin, abnormal functioning of the immune system, pregnancy, coagulopathy, and liver failure.

### 2.3. Characteristics of Surgery Conditions

The body temperature of the patients was maintained at 33–36 °C, depending on the type of surgery. The use of CPB was preceded by the administration of heparin at a dose of 3 mg/kg of body mass to ensure that the activated clotting time was longer than 480 s, in contrast to that before the surgery (100–120 s). In the case of cardiac arrest, cold (4 °C) cardioplegia, consisting of Plasmalyte supplemented with 15% potassium chloride and 15% mannitol, was applied to the aortic bulb. At the end of the surgery, the electrical and mechanical functions of the heart returned spontaneously due to the restoration of coronary circulation after aortic declamping.

### 2.4. Collection and Preparation of Samples

Blood samples (2.6 mL) were routinely collected from the patients at each of the following time points: (1) before the use of CPB, (2) after 60 min of the use of CPB, and (3) at 24 h after the beginning of the surgery. The collected blood samples were centrifuged at 1200× *g* for 10 min. The resulting serum and plasma fractions were stored at −20 °C until further analyses.

The concentration of the IgG antibody was measured by a competitive ELISA (enzyme linked immunosorbent assay) diagnostic test (CUSABIO, Wuhan, China). Direct ELISA diagnostic tests were applied for determining the concentrations of IgA (AccuDag, Beijing, China), IgD (Innovative Research, Novi, MI, USA), and IgM (CUSABIO, Wuhan, China), while indirect ELISA diagnostic tests were performed for measuring the concentrations of IgG3 (Abnova, Taipei, Taiwan) and IL-6 (RayBio, Peachtree Corners, GA, USA). Antibody-coated 96-well plates were used for ELISA tests, according to the manufacturer’s instructions. For each experiment, the samples were analyzed in duplicate, and a new standard curve was plotted using CurveExpert Professional software (CurveExpert Pro 2.3.0 started, with process ID 4648). Absorbance was measured using a Dynex Opsys MR Microplate Reader (Dynex Technologies, Chantilly, VA, USA) after its systematic validation for clinical analyses.

### 2.5. Statistical Analyses

Statistical analyses were performed using STATISTICA 13 software (Statsoft, Kraków, Poland). Descriptive statistics were used, and the data obtained for each group were evaluated by Shapiro–Wilk test. Statistical analyses of the differences between age groups and genders were performed using Mann–Whitney *U* test. The differences among the results of immunoenzymatic assays performed at different time points were analyzed using Friedman’s test [25]. All differences were considered significant at *p* < 0.05.

## 3. Results

Six males and seven females aged 19–80 years were chosen for the analysis of IL-6 and IgA concentrations, and seven males and six females aged 19–80 years were selected for the analysis of IgG, IgM, IgD, IgE, and IgG3 concentrations (first patient in 2011, last patient out 2012) (Table 1). The selection criteria for inclusion and exclusion from the therapy (point 2.2) and the randomization process resulted in such a small number of patients enrolled for the analysis. The selected patients were assigned to two subgroups according to age: 19–46 years and 73–80 years. Additionally, a group of patients with a diagnosed postoperative infection was distinguished (Table 2).

### 3.1. IL-6

**Figure 1:**
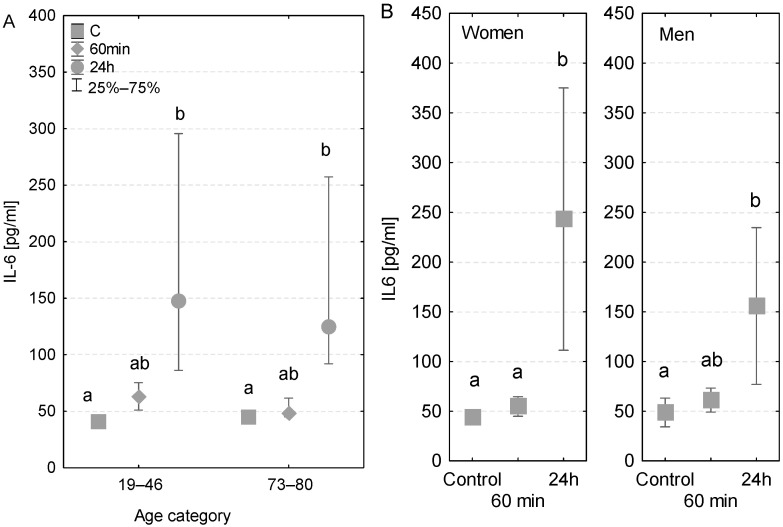
Serum IL-6 concentration (median ± quartile deviation; 25th and 75th percentiles). (**A**) Comparison based on the age of patients: 19–46 years and 73–80 years. (**B**) Comparison based on the sex of the patients. CT: measurement before the onset of CPB; 60 min: measurement at 60 min of the CPB onset; 24 h: measurement at 24 h after the onset of CPB. Values with different letters (a, b) indicate significant differences within the age or sex category (Friedman’s test or Mann–Whitney U test; *p* < 0.05).

The measured concentrations of immunological parameters (50–300 pg/mL) greatly exceeded the previously published reference values, which were <10 pg/mL. The levels of IL-6 found in younger and older patients were analogous. In both age groups, no major changes in concentrations were detected at 60 min of the CPB onset. However, a 1.4-fold significant increase in IL-6 was noted among younger patients and a 1.6-fold increase among older patients after the first postoperative day (Figure 1A). Furthermore, in the case of older patients, the concentration of IL-6 measured at 24 h was significantly higher than that measured at 60 min. An increased level of IL-6 was observed in the patient who was diagnosed with a postoperative infection after the first postoperative day (318 pg/mL) compared to the value measured at 60 min of the CPB onset (52 pg/mL) and the control value measured before the surgery (49 pg/mL, Table 3).

The concentrations of IL-6 measured before the surgery were almost identical in both genders (43.7 and 43.1 pg/mL, respectively) (Figure 1B). No significant differences were found in levels measured at 60 min of the CPB onset in terms of both gender and sex. However, after the first postoperative day, a 1.3-fold significant increase in the level of IL-6 was observed in the group of men and a 2.8-fold significant increase among women (Figure 1B), compared to the control levels.

### 3.2. IgG

**Figure 2:**
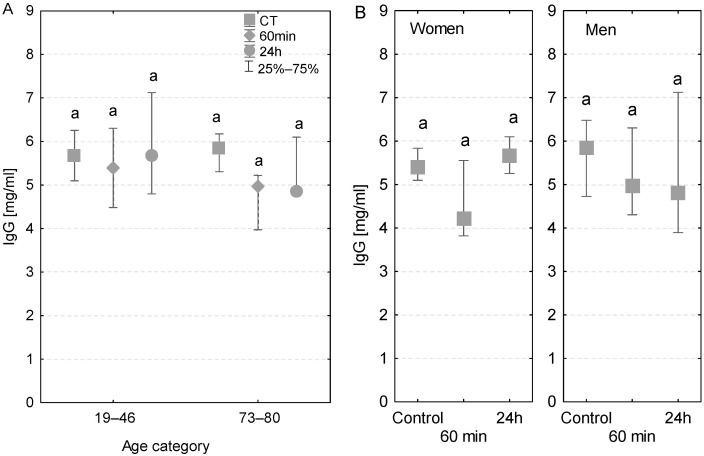
Serum IgG concentration (median ± quartile deviation; 25th and 75th percentiles). (**A**) Comparison based on the age of patients: 19–46 years and 73–80 years. (**B**) Comparison based on the sex of the patients. CT: measurement before the onset of CPB; 60 min: measurement at 60 min of the CPB onset; 24 h: measurement at 24 h after the onset of CPB. The same letters (a) indicated no statistical differences within age or sex category (Friedman’s or Mann–Whitney U test; *p* < 0.05.).

Regardless of the age group, gender, time of measurement, and individual cases of infection, the serum levels of IgG in patients were lower than the reference values ranging between 8 and 16 mg/mL (Figure 2A,B). Additionally, after 24 h, the serum IgG concentration in women was higher than that in men. In patients with no postoperative infections, there were no significant differences in serum IgG concentrations measured at different time points. However, in the group of elderly patients, with and without postoperative infections, there was a decline in the serum IgG concentration measured at 60 min and 24 h (Figure 2A). Among patients who had postoperative infections, the serum IgG concentration measured at 60 min of the CPB onset was lower compared to the control values. At 24 h after the CPB onset, the IgG concentrations individually varied in patients diagnosed with infection (Table 3).

### 3.3. IgM

**Figure 3:**
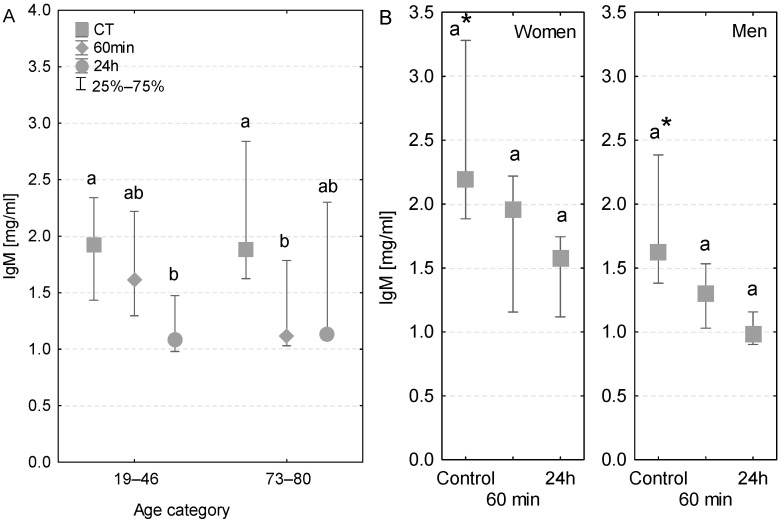
Serum IgM concentration (median ± quartile deviation; 25th and 75th percentiles). (**A**) Comparison based on the age of patients: 19–46 years and 73–80 years. (**B**) Comparison based on the sex of the patients. CT: measurement before the onset of CPB; 60 min: measurement at 60 min of the CPB onset; 24 h: measurement at 24 h after the onset of CPB. Values with different letters (a, b) indicate significant differences within the age or sex category (Friedman’s test or Mann–Whitney U test; *p* < 0.05); * indicates statistical difference between women and men; *p* < 0.05.

The level of serum IgM antibody was within the reference range (0.5–2 mg/mL). A significant decrease in IgM concentration was observed in the serum of younger patients after the first postoperative day (Figure 3A). In the case of older patients, the serum IgM concentration was significantly lower even at 60 min of the CPB onset (Figure 3A).

In control conditions, the IgM concentration was nearly 2-fold higher in the serum of women than that in men. At both 60 min and 24 h, the level of IgM was lower in the serum of both women and men in comparison to control conditions (Figure 3B). At 60 min of the CPB onset, the IgM concentration was lower in the serum of both patients with postoperative infections. However, at 24 h, only one of the infected patients had lower serum IgM concentration, whereas in the second patient the IgM value returned to the control level (Table 3).

### 3.4. IgD

**Figure 4:**
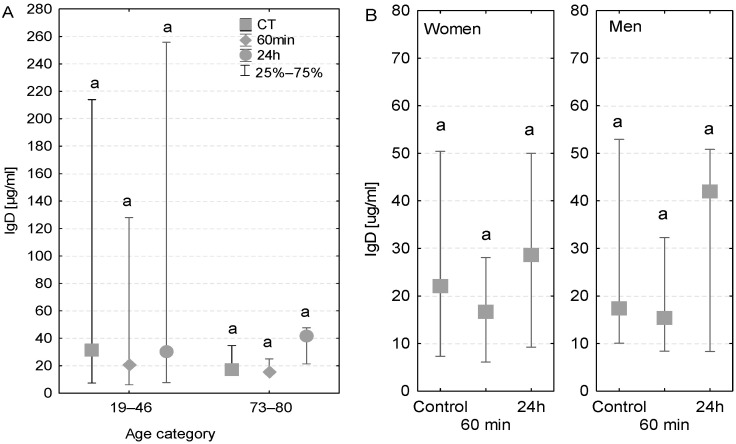
Serum IgD concentration (median ± quartile deviation; 25th and 75th percentiles). (**A**) Comparison based on the age of patients: 19–46 years and 73–80 years. (**B**) Comparison based on the sex of the patients. CT: measurement before the onset of CPB; 60 min: measurement at 60 min of the CPB onset; 24 h: measurement at 24 h after the onset of CPB. The same letters (a) indicated no statistical differences within age or sex category (Friedman’s or Mann–Whitney U test; *p* < 0.05.).

The concentration of IgD antibodies measured in the blood serum of the majority of examined patients was close to the reference value (40 µg/mL). However, among younger patients, a wide variation in IgD levels was observed, which can be explained by the fact that two patients had higher IgD levels than the reference value (128–412 µg/mL, Figure 4A). In the case of elderly patients, a similar pattern was observed in both patients with and without diagnosed postoperative infection (Table 3). More precisely, the IgD concentrations in these groups were higher than the control value after the first postoperative 24 h (33 µg/mL compared to 20 µg/mL in control conditions, Figure 4A). No major differences in the level of IgD antibodies were observed between women and men (Figure 4B).

### 3.5. IgE

**Figure 5:**
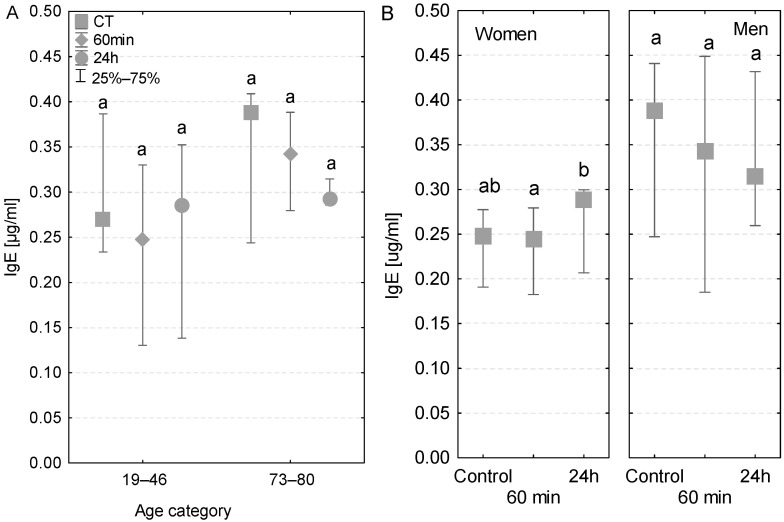
Serum IgE concentration (median ± quartile deviation; 25th and 75th percentiles). (**A**) Comparison based on the age of patients: 19–46 years and 73–80 years. (**B**) Comparison based on the sex of the patients. CT: measurement before the onset of CPB; 60 min: measurement at 60 min of the CPB onset; 24 h: measurement at 24 h after the onset of CPB. Values with different letters (a, b) indicate significant differences within the age or sex category (Friedman’s test or Mann–Whitney U test; *p* < 0.05).

In the two different age groups, the serum IgE concentrations were within the range of reference values (0.017–0.45 µg/mL). In the group of older patients, the IgE levels tended to decrease gradually at subsequent sampling time points and reached the lowest value at 24 h after the beginning of the operation (Figure 5A). Conversely, this tendency was not observed among younger patients or in patients with postoperative infections (Table 3). In terms of gender, an increase in IgE concentrations was observed in women (Figure 5B) and a decrease was observed in men. Nevertheless, the level of this antibody was quite lower in women compared to men. Interestingly, only in the case of women was the increase in IgE concentration observed at 24 h significant compared to the sample taken during the operation (Figure 5B).

### 3.6. IgA

**Figure 6:**
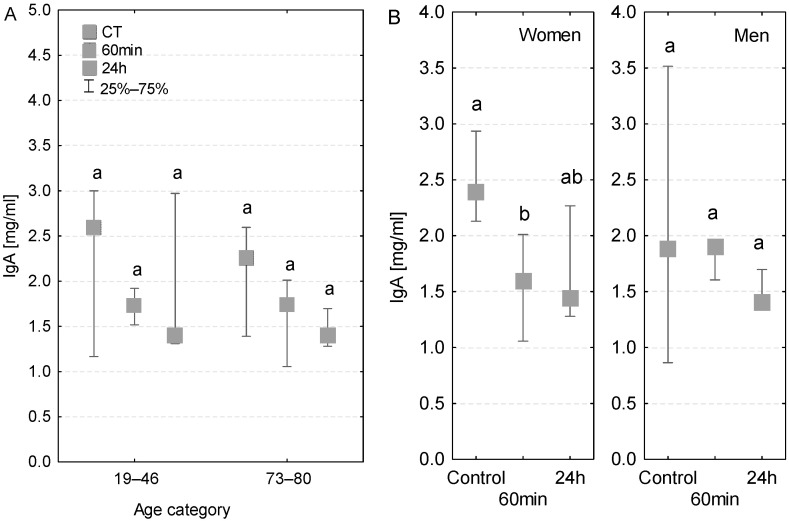
Serum IgA concentration (median ± quartile deviation; 25th and 75th percentiles). (**A**) Comparison based on the age of patients: 19–46 years and 73–80 years. (**B**) Comparison based on the sex of the patients. CT: measurement before the onset of CPB; 60 min: measurement at 60 min of the CPB onset; 24 h: measurement at 24 h after the onset of CPB. Values with different letters (a, b) indicate significant differences within the age or sex category (Friedman’s test or Mann–Whitney U test; *p* < 0.05).

The IgA concentrations measured in the analyzed age groups were generally within the reference range (1–3 mg/mL). The concentrations of IgA antibodies measured in serum at both 60 min of the CPB onset and 24 h after the beginning of the surgery were lower compared to the control measurements. The level of IgA antibodies was also within the reference range in the serum of patients with diagnosed postoperative infection (Table 3), but in this group the values decreased in subsequent time points (Figure 6A). In women, the decrease in the level of IgA class antibodies was statistically significant at 60 min of the CPB onset (Figure 6B). At 24 h, the serum IgA concentration was even lower, but the decrease was not significant. In the case of men, the serum IgA concentration at 60 min of the CPB onset remained similar to the control level, whereas on the first postoperative day it was slightly lower.

### 3.7. IgG3

**Figure 7:**
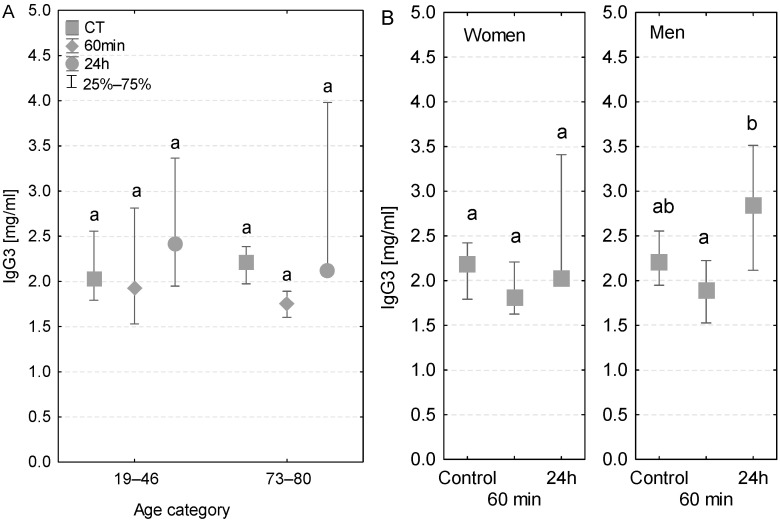
Serum IgG3 concentration (median ± quartile deviation; 25th and 75th percentiles). (**A**) Comparison based on the age of patients: 19–46 years and 73–80 years. (**B**) Comparison based on the sex of the patients. CT: measurement before the onset of CPB; 60 min: measurement at 60 min of the CPB onset; 24 h: measurement at 24 h after the onset of CPB. Values with different letters (a, b) indicate significant differences within the age or sex category (Friedman’s test or Mann–Whitney U test; *p* < 0.05).

Both age groups had a similar pattern of changes in IgG3 concentration—lower values during the surgery, followed by a rise after the first postoperative day (Figure 7A). However, no statistically significant differences were detected between the groups of younger and elder patients. A similar tendency was observed in patients with diagnosed postoperative infection (Table 3). In addition, the pattern of changes in the IgG3 level measured in women and men was also similar (Figure 7B). However, in men, the increase in IgG3 concentration measured after 24 h was statistically significant (Figure 7B).

## 4. Discussion

This study provides comparative data regarding fluctuations in the levels of IL-6 and immunoglobulins in the serum of patients undergoing cardiac operations supported by CPB, depending on their age and gender. Regardless of the patients’ age, the serum IL-6 concentration was significantly higher on the first postoperative day than that measured before the surgery. This finding is consistent with the findings of the study by Gozdzik et al., which examined the level of serum IL-6 in patients undergoing cardiac surgery with the use of CPB [26]. The authors observed that the concentration measured at 6 h and at 24 h after the surgery was 52.2 pg/mL and it was dramatically higher, by nearly 10-fold, than the value measured before anesthesia (5.1 pg/mL).

Greenberg et al. [16], who analyzed the serum concentration of IL-6 in children undergoing surgery supported by CPB, concluded that this cytokine may serve as a marker of infection. According to their hypothesis, the IL-6 marker would be useful in the selection of patients and for minimizing possible postoperative organ dysfunctions associated with the CPB technique [13]. Furthermore, Diegeler et al. compared the levels of IL-6 in patients undergoing various types of surgery, such as operations supported by CPB, off-pump coronary artery bypass surgery, and minimally invasive direct coronary artery bypass surgery [27]. Their study revealed that regardless of the type of operation, a similar tendency was observed in IL-6 concentrations measured at subsequent time points. However, the level of IL-6 measured after 24 h of the CPB-supported surgery was significantly higher than that measured after the other two minimally invasive types. During anesthesia, the level of IL-6 or other markers, such as IL-8, IL-10, TNF-R p75, and C5a, did not show statistically significant changes. The use of the CPB technique induced a perioperative increase in the level of the analyzed markers, which remained high (except C3d) even in the postoperative period. This suggests that the type of operation may determine whether the elicited immune response is humoral or cellular [27]. Moreover, activation of the complement cascade is observed, accompanied by an increase in the levels of inflammatory regulators such as IL-6, IL-8, and TNF, leading to the release of leukocytes from the bone marrow [28]. He et al. hypothesized that SIRS with bacterial or other etiology is a characteristic feature of surgeries involving the CPB technique [29]. It has been shown that the serum IL-6 concentration decreases with age, and that this effect is more pronounced in females than in males [30]. Oertelt-Prigione et al. investigated the effect of estrogen on the function of the immune system [31]. However, these authors did not find any correlation between the levels of estrogen, progesterone, or testosterone and the amount of cytokines secreted by leukocytes. Menstrual cycle, and menopause in the case of elderly females, can complicate the analysis of the relationship between gender and immunity. Nevertheless, the distinct functioning of the immune system in females and males may be partially explained by the potential regulatory effect of estrogen on the cytokine secretion by immune cells.

Despite multiple studies, it remains unclear whether gender has an influence on the functioning of the immune system. However, one must consider the impact of the aging process, which progresses differently in females than males. For instance, reactive oxygen species are produced at a lower level in women than in men, as estrogens, mainly estradiol, play a protective role. The protective mechanism of action of steroid hormones can probably be related to the regulation of the expression of genes encoding antioxidant enzymes [32]. Furthermore, estrogens have been shown to reduce the amount of serum lipids, and thus lower the risk of cardiovascular diseases. Another fact supporting the protective role of estrogen is the increased incidence of cardiovascular diseases in postmenopausal women [33]. Steroid hormones that bind to specific receptors can change the behavior of immune cells. Estrogen receptors are expressed not only in gametes but also on the surface of lymphocytes, monocytes, or macrophages. In general, an increase in estrogen intensifies the immune response; however, this effect may be inhibited by progesterone and androgens. Estradiol also indirectly influences the immune response by activating the MAPK and NF-κB signaling pathways that regulate the expression of genes encoding antioxidant enzymes. Thus, women have a lower level of reactive oxygen species than men [31]. However, it is unclear whether the immune response significantly differs between female and male patients undergoing cardiac surgery with the use of CPB. In 1994, Fernandez et al. conducted a large study with 1200 patients which revealed increased morbidity and mortality in female patients undergoing cardiac surgery supported by CPB [34]. However, at the time of this study, the hospital standards were outdated, and the surgical techniques were not very reliable. In 1997, Rinder et al. examined the presence of a correlation between the gender of patients and impaired immune response due to CPB [35]. The authors observed several dysregulations depending on the age of patients on the first three postoperative days; however, no relationship between the gender and the amount of immune cells produced was observed.

In the present study, a significantly higher concentration of IgM antibodies was observed in the serum of female patients in comparison to males. This is consistent with the previous findings of Oertelt-Prigione, who demonstrated the stimulating effect of estrogens and the inhibitory effect of testosterone on the production of IgM antibodies by plasma cells in women of reproductive age [31]. Studies have shown fluctuations in the level of serum immunoglobulins both during the operation and in the first days of the postoperative period [20,21]. These observations cannot be explained only by the fact that CPB results in partial dilution of patients’ blood. Tajima et al. [4] analyzed the serum levels of IgG, IgA, and IgM antibodies in patients after cardiac operation supported by CPB [4]. Although the authors observed a postoperative decrease in the level of antibodies, the differences in the frequency of postoperative infections were not statistically significant. Similarly, Lante et al. [17] analyzed the concentrations of IgE, IgM, and IgG1–4 antibodies. These authors observed that the IgE levels did not change until postoperative day 3 but increased significantly on postoperative day 5, while the levels of both IgG and IgM decreased significantly on postoperative day 1. The level of IgM returned to the baseline value on postoperative day 5, whereas IgG remained below the baseline level until postoperative day 5 [17]. The above authors also calculated the IgG2/IgE ratio to determine the direction of the developing immune response. Their results showed the predominance of IgE at each measuring point, which indicated a shift in the immune response toward humoral reaction [21]. On the other hand, the increased level of IgG2 antibodies seemed to indicate a Th1-dependent response [36]. Based on their findings, Lante et al. concluded that the observed result is a consequence of extensive, prolonged surgery [17]. This indicates that changes in antibody concentration associated with the use of CPB may mostly depend on the class of antibody. The present study showed a distinct immune response in younger patients in comparison to older patients, which suggests noninfectious recuperative recovery. Only for the IgM class of antibodies, significant differences were noted in concentration depending on the patient age and sampling time. In younger patients, the concentration of IgM was significantly lower after the first postoperative day, whereas in elderly patients a significant decrease was noted in the samples taken during the operation. Due to the low specificity of the IgM antibody, changes in its concentrations observed in both age groups of patients may reflect impaired defense mechanisms against pathogens during the early stages of infection. This study attempted to search for a new marker that could detect postoperative infections early. In addition, our study indicates differences in antibody concentrations between female and male patients undergoing cardiac surgery with CPB. However, considering sex differences, a greater number of patients should be recruited to draw definitive conclusions.

## 5. Conclusions

The impact of the CPB technique on the quality of cardiac surgeries and the serum level of immunological parameters such as IL-6 must be studied further to understand the impairment of immune function by SIRS. Undoubtedly, the number of patients who are qualified for cardiac surgery with the use of CPB is increasing in the aging population. Therefore, further trials are necessary to assess whether the type of changes occurring in the parameters of the immune system due to the use of CPB could make a patient susceptible to infections in the postoperative period. The identification of factors that can increase the risk of postoperative bacterial infections is crucial to accelerate the recovery rate of cardiac patients and, in the long run, to likely exclude the risk of infections resulting from CPB.

## Figures and Tables

**Table 1:** Demographic data of patients selected for the analyses.

IL-6 and IgA Concentration Analyses						
Group of Patients	Parameter	Age (Years)	EuroSCORE	BMI	Circulation Duration (min)	Aortic Cross-Clamping Duration (min)
Women	Average	61	7	26.8	132	82
	Min	19	5	20	95	58
	Max	83	12	33	171	120
	Number of patients	6				
Men	Average	55	6	25.2	98	67
	Min	26	3	20	59	34
	Max	83	8	32	157	124
	Number of patients	6				
Patients at the age of 18–69	Average	39	7	23.3	121	74
	Min	19	3	20	59	34
	Max	61	12	30	157	124
	Number of patients	6				
Patients at the age of 70 and above	Average	77	7	28.7	112	76
	Min	72	5	24	71	39
	Max	83	8	33	171	120
	Number of patients	6				
IgG, IgM, IgD, IgE, and IgG3 concentration analyses						
Women	Average	51	6	30.2	104	62
	Min	21	3	24	80	44
	Max	76	9	39	121	80
	Number of patients	5				
Men	Average	49	4	25.7	154	100
	Min	19	2	19	104	64
	Max	76	6	33	307	235
	Number of patients	6				
Patients at the age of 18–69	Average	30	4	28.5	139	96
	Min	19	2	19	80	57
	Max	46	6	39	307	235
	Number of patients	6				
Patients at the age of 70 and above	Average	75	7	26.8	114	66
	Min	73	5	22	104	44
	Max	76	9	33	123	76
	Number of patients	5				

**Table 2:** Data of patients (P1, P2, and P3) with a diagnosed postoperative infection.

Characteristic	P1	P2	P3
Sex	Man	Woman	Woman
Age (years)	73	80	77
EuroSCORE	12	7	8
BMI	28	23	22
Circulation duration (min)	368	100	147
Aortic cross-clamping duration (min)	207	62	105

**Table 3:** Concentrations of immunoglobulins and IL6 in sera of patients with a diagnosed postoperative infection: P1 (men), P2, and P3 (women).

Time Point	IgG (mg/mL)		IgG3 (mg/mL)		IgM (mg/mL)		IgD (µg/mL)		IgE (µg/mL)		IgA (mg/mL)	IL6 (pg/mL)
	Patient											
	P1	P2	P1	P2	P1	P2	P1	P2	P1	P2	P3	P3
CT	5.02	4.39	3.09	2.42	2.38	1.83	26	9.38	0.25	0.25	2.87	49.01
60 min	4.31	3.75	1.96	1.86	1.53	1.16	15.9	8.2	0.19	0.23	2.05	52.26
24 h	3.89	5.25	2.29	3.41	0.98	1.75	44.68	9.25	0.35	0.21	1.89	318.29

## Data Availability

Data available on request due to restrictions privacy or ethical.
